# Judgment aggregation, discursive dilemma and reflective equilibrium: Neural language models as self-improving doxastic agents

**DOI:** 10.3389/frai.2022.900943

**Published:** 2022-10-18

**Authors:** Gregor Betz, Kyle Richardson

**Affiliations:** ^1^Karlsruhe Institute of Technology, Department of Philosophy, Karlsruhe, Germany; ^2^Allen Institute for Artificial Intelligence, Aristo, Seattle, WA, United States

**Keywords:** neural language model (NLM), judgment aggregation, reflective equilibrium, text generation, logical consistency

## Abstract

Neural language models (NLMs) are susceptible to producing inconsistent output. This paper proposes a new diagnosis as well as a novel remedy for NLMs' incoherence. We train NLMs on synthetic text corpora that are created by simulating text production in a society. For diagnostic purposes, we explicitly model the individual belief systems of artificial agents (authors) who produce corpus texts. NLMs, trained on those texts, can be shown to aggregate the judgments of individual authors during pre-training according to sentence-wise vote ratios (roughly, reporting frequencies), which inevitably leads to so-called discursive dilemmas: aggregate judgments are inconsistent even though all individual belief states are consistent. As a remedy for such inconsistencies, we develop a self-training procedure—inspired by the concept of reflective equilibrium—that effectively reduces the extent of logical incoherence in a model's belief system, corrects global mis-confidence, and eventually allows the model to settle on a new, epistemically superior belief state. Thus, social choice theory helps to understand why NLMs are prone to produce inconsistencies; epistemology suggests how to get rid of them.

## 1. Introduction

Statistical language models describe the probability distribution of tokens (e.g., words) in a language (Manning and Schütze, 1999). Technological advances in the design of neural networks have recently led to the development of powerful machine learning models, so-called Transformers (Vaswani et al., 2017), which predict language tokens with previously unseen accuracy and have since sparked a scientific revolution in the field of AI and NLP: These neural language models (NLMs)—such as GPT-2 (Radford et al., 2019) and GPT-3 (Brown et al., 2020), BERT (Devlin et al., 2019) and RoBERTa (Liu et al., 2019), or T5 (Raffel et al., 2020)—are not only regularly achieving ever better SOTA results in traditional NLP tasks like machine translation, reading comprehension, or natural language inference (as documented, e.g., on paperswithcode.com); they are also successfully applied to solve further cognitive tasks involving advanced reasoning, specifically multi-hop inference (Clark et al., 2020; Saha et al., 2021), explanation (Yang et al., 2018; Zaheer et al., 2020; Dalvi et al., 2021), creative writing (Holtzman et al., 2019), commonsense reasoning (Bosselut et al., 2019b), critical thinking (Betz et al., 2021a), or mathematical theorem proving (Polu and Sutskever, 2020; Noorbakhsh et al., 2021). These broad and robust predictive successes naturally trigger the questions (i) whether it makes sense— conceptually and normatively—to say that NLMs exhibit human rationality (cf. Zimmermann, 2020), and (ii) whether NLMs represent empirically adequate models of human cognition (cf. Goldstein et al., 2020; Schrimpf et al., 2021).

However, and despite their revolutionary impact, NLMs still face important limitations. Arguably one of their major, widely acknowledged failures consists in the fact that the output of NLMs suffers from spectacular inconsistencies (Ribeiro et al., 2019; Ettinger, 2020; Kassner and Schütze, 2020). For example, XLM-Roberta (Conneau et al., 2019) judges that Warsaw lies north of Berlin, Berlin north of Paris, and Paris north of Warsaw^1^. Likewise, Delphi (Jiang et al., 2021b) ponders that it's wrong to hurt the cat (or the dog) so that the dog (respectively, the cat) can survive, yet that it's equally wrong to let both cat and dog die^2^. In this paper, we argue that the emergence of such inconsistencies might be partially explained in terms of *judgment aggregation* during the model's pre-training, and we introduce, moreover, a novel *self-contained, self-improving* fine-tuning procedure which effectively reduces global inconsistencies.

Let us for a moment conceive of judgment, or belief, as a binary classification task: a sentence is classified as either true or false. Given that NLMs—qua learning objective—seek to match the token distribution of the training data, it seems highly plausible that a NLM's confidence in its classification of sentence *s* as true correlates closely with the relative frequency of *s* being presented as true (rather than false) in the training data. In this perspective, we may expect NLMs to aggregate judgments (from the training data) sentence-wise and in accordance with vote ratios (assuming, for now, each training text has one vote).

The hypothesis of sentence-wise vote ratio aggregation, albeit plausible and predictable, has surprising consequences. It is a well-known result from social choice theory that aggregating a profile of *individually consistent* sets of judgments by means of sentence-wise majority vote may result in an *inconsistent* set of *collective* judgments—if, and only if, some judgments range over a minimally inconsistent set of sentences of length equal to or greater than three (see List, 2013). This phenomenon, which mirrors Arrow's impossibility theorem for preference aggregation (Arrow, 1951), is also referred to as **discursive dilemma** (Pettit, 2001). Now, provided that neural language models form judgments in accordance with sentence-wise vote ratio aggregation, we shouldn't be surprised to find that these judgments are logically inconsistent, even if all the *training texts* are individually consistent. Discursive dilemma hence provides a potential explanation for why a language model makes inconsistent judgments. We will quantify the extent of such judgment-aggregation-induced incoherence.

Can a neural language model get rid of the inconsistencies in its belief system which have arisen from discursive dilemmas? We propose a method for doing so. The key idea is to let the neural language model go through a process of gradual belief revision, inspired by the concept of reflective equilibrium. Reflective equilibrium has been originally introduced by the eminent philosophers Nelson Goodman and John Rawls as a method for how normative beliefs are formed, rationally revised, and justified (Goodman, 1955; Rawls, 1971). It has since been extensively discussed and refined (e.g., Daniels, 1996; Brun, 2014; Baumberger and Brun, 2016; Elgin, 2017), and is today arguably one of the major views about rational belief formation in ethics, logic, philosophy, and epistemology. For all its prominence and despite several formal explication attempts (Tersman, 1993; Thagard, 2000; Yilmaz et al., 2016; Beisbart et al., 2021), there is no agreement about what *exactly* this method amounts to. We conceive of **reflective equilibrium**, for the purposes of this paper, as a process of step-wise and local belief revision, where

[RE-process 1] each modification is triggered by a critical logical assessment of a finite (typically small) sub-part of the entire current belief system;
[RE-process 2] step-wise adjustments seek to locally improve the mutual justification (logical fit) between individual beliefs;

with the overarching aims:

[RE-aim 1] in the long run, the continuous revisions logically improve (e.g., increase global coherence of) the belief system as a whole;
[RE-aim 2] the evolving belief system converges toward a new belief state.

Such a thin conception of reflective equilibrium resembles connectionist accounts of coherence, proposed in philosophy (Thagard, 1992, 2000) and psychology (Simon et al., 2004, 2015). We may note, however, that it differs fundamentally from Bayesian updating (Jeffrey, 1965), AGM belief revision (Alchourron et al., 1985), or formal learning theory (Kelly, 1996) inasmuch as beliefs are not *required* to be logically consistent from the outset, and may be revised *without* external triggers such as the acquisition of novel facts or evidence.

This paper's attempt to emulate advanced *normative* theories of rational agency (namely, the theory of reflective equilibrium) with and through NLMs is in line with recent *empirical* findings in cognitive science which establish that NLMs, and in particular Transformers, can explain both the behavioral and the neural response of the human brain in high-level language processing tasks (Goldstein et al., 2020; Schrimpf et al., 2021).

**Figure 1:**
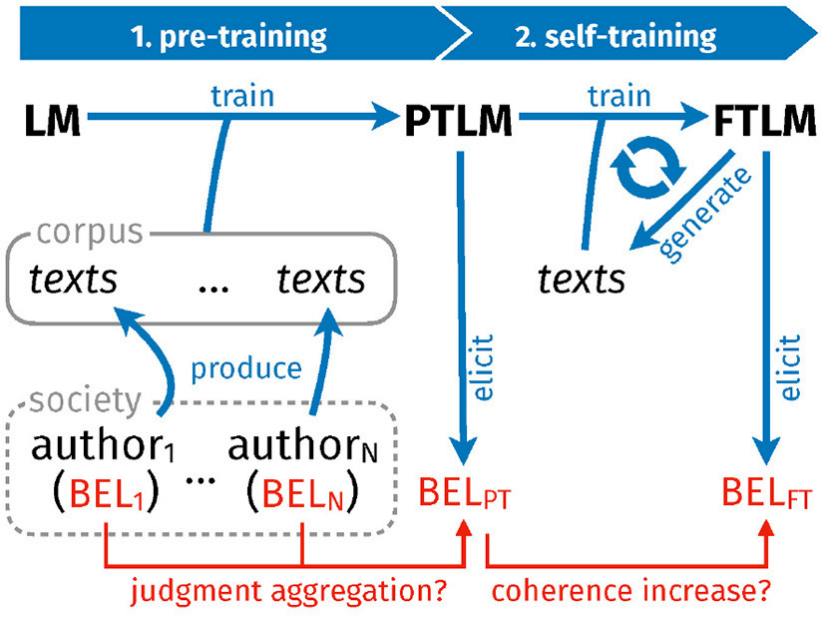
Overall design. A randomly initialized language model (LM) is trained on a synthetic text corpus produced by artificial, simulated authors. The accordingly pre-trained model (PTLM) subsequently trains on self-generated texts, which finally yields a fine-tuned model (FTLM).

Figure 1 presents the overall design of our specific computational experiments, which fall in two parts. In part one (*pre-training*), we train randomly initialized Transformer language models on carefully constructed text corpora (cf. Section 3.2). Each text corpus is built by simulating a society of authors who hold (internally consistent) beliefs about how to sort items in a domain, and express their views in argumentative texts (cf. Section 3.1). To further increase experimental control and to eliminate confounding factors (e.g., tokenization), texts are composed in a simple and transparent *artificial language*, rather than a natural one. (Consequently, the Transformer learns but the artificial language.) The artificial language has a straight-forward semantic interpretation: One may use it to articulate a strict order in a domain. Now, by eliciting the degrees of belief of the pre-trained language models and comparing those with the beliefs of the simulated authors who have produced the training texts in the first place (cf. Section 3.4), we examine the language models' belief formation mechanism and the extent of judgment-aggregation-induced inconsistencies (i.e., the output inconsistency that can be explained with reference to the model's specific way of aggregating judgments).

In part two of the experiments (*self-training*), we submit the pre-trained language models to continuous self-training. More specifically, a model generates, at each step of the self-training loop, a series of texts which are supposed to spell out the logical implications of a small subset of the model's current beliefs (we prompt the model with sentences it tends to consider true). The generated texts—which may be conceived as a model's simple “self-reflections” and attempts to locally “think through” its current beliefs—are processed and transformed into suitable training data on which the model is eventually trained (cf. Section 3.3). Accordingly defined self-training, with sub-steps (i) text-generation and (ii) training on self-generated texts, corresponds closely to properties [RE-process 1] and [RE-process 2], which characterize reflective equilibrium. Tracking the evolution of the models' belief systems during self-training, we assess whether inconsistencies are resolved and the model converges toward an improved belief state (cf. Section 3.5). In other words, we verify whether the process is conducive to [RE-aim 1] and [RE-aim 2]. As a simple baseline, we consider an analogous fine-tuning loop where texts are picked from the original corpus rather than generated by the model, but which is, otherwise, identical to the self-training loop.

The main findings of these experiments can be summarized as follows:

(R1) Neural language models trained on an unbiased text corpus compiled by a group of authors form beliefs, *grosso modo*, in accordance with sentence-wise vote ratios (one author, one vote); especially so if the number of authors who withhold their judgment is small (Cf. Section 4.1).
(R2) Pre-trained language models may exhibit judgment-aggregation-induced inconsistencies. Both the frequency and the gradual severity of logical inconsistencies in the models' belief systems correspond closely to those observed in the underlying societies' collective beliefs (i.e., vote ratios) (Cf. Section 4.1).
(R3) Training on self-generated texts substantially reduces the extent of logical inconsistencies and hence improves the coherence of the models' belief systems (Cf. Section 4.2). The fact that self-generated texts (i) are inferentially structured and (ii) occasionally contain sentences the model actually disbelieves (at the time when generating the text) suggests that the observed coherence improvements are brought about by a rational belief revision mechanism.
(R4) Pre-trained models are mostly over-confident, in the sense that their degrees of belief are globally more informative than the collective beliefs of the authors (vote ratios). Such initial mis-confidence is effectively reduced through self-training, giving rise to a characteristic pattern (sharp initial drop followed by a gradual build-up of informativeness) (Cf. Section 4.3).
(R5) The belief dynamic's volatility decreases sharply during self-training, and overall divergence from the initial belief state doesn't rise any further from a given point onwards. That is, each model's belief system converges to a new equilibrium state. Moreover, the more coherent the pre-trained model's beliefs are in the first place, the less it diverges from its initial belief state during self-training (Cf. Section 4.4).

We consider these to be significant results which altogether justify the conclusion that the language models we study in a synthetic environment rationally self-improve their belief states by undergoing a process of reflective equilibration, as they meet the conditions [RE-process 1], [RE-process 2], [RE-aim 1], and [RE-aim 2].

While our experiments suggest a novel explanation for, and a potential remedy against the tendency of large language models such as GPT-3 or T5 to generate globally inconsistent output, it is still an open question to which extent (a) those inconsistencies in fact stem from judgment aggregation effects and (b) models like GPT-3 or T5 can actually self-improve by reflective equilibration. The very same simplifying assumptions which allow us to study belief formation processes in NLMs by means of computational experiments (in particular the extremely simple artificial language) weaken the analogy to models trained on natural languages. These limitations of the current study call for follow-up investigations and may open up fruitful research perspectives (cf. Section 5).

## 2. Related work

### 2.1. Accuracy and consistency of NLMs' factual knowledge claims

Pre-trained language models have been found to be rich and—to a certain extent—accurate knowledge bases (Petroni et al., 2019; Radford et al., 2019). Da et al. (2021) demonstrate that fine-tuning on knowledge graph data (Bosselut et al., 2019a,b) is a particularly effective way for eliciting such commonsense judgments. Knowledge extraction is, however, tricky. Judgments elicited from a model are highly context sensitive (Petroni et al., 2020), prone to (mis-)priming effects (Kassner and Schütze, 2020) and tend to be collectively inconsistent (Ribeiro et al., 2019; Elazar et al., 2021; Jang et al., 2021). Symptomatically, pre-trained language models struggle with negation (Ettinger, 2020; Kassner and Schütze, 2020; Talmor et al., 2020; Jiang et al., 2021a). In an investigation that methodologically parallels this paper's approach, Kassner et al. (2020) study belief formation of NLMs by pre-training a model on a synthetic, logically structured symbolic text corpus. Kassner et al. (2020) observe that belief formation is mainly triggered by memorization effects (rather than reasoning) and is strongly determined by the text frequencies of the corresponding facts. Our findings on belief formation as judgment aggregation are consistent with those results (see also the discussion of mis-calibration below).

As a remedy for incoherence, Kassner et al. (2021a,b) propose to add extra-architecture to a NLM for ensuring that globally consistent beliefs can be elicited from the structurally expanded system. In a similar vein, and drawing from cognitive dual process theories (Kahneman, 2011), Nye et al. (2021) interpret NLMs as fast yet error-prone systems, and demonstrate that these may be complemented by lightweight symbolic processes to increase the global consistency of the output. Recent work on perceptual grounding of NLMs shows that the integration of NLMs into a global neural architecture which interacts with a physical environment increases the ability of NLMs to correctly predict *entire, complex* physical situations (Zellers et al., 2021), which, in turn, seems to suggest that perceptual grounding fosters global *consistency* (of complex NLM output) as well.

Finding that the beliefs of our pre-trained language models are logically inconsistent, our study agrees with the literature reported above. However, we go beyond the literature in suggesting (i) a novel (additional, rather than rival) explanation for the NLMs' logical incoherence, namely discursive dilemmas, and in devising (ii) a self-training regime that effectively resolves these sort of inconsistencies. In particular, our self-training process does not rely on external computational resources, but is *self-contained*: it just makes use of the linguistic abilities available to the NLM itself.

### 2.2. Mis-calibration and over-confidence of NLMs

Guo et al. (2017) observe that modern, deep networks used for classification tasks are, in general, poorly calibrated, i.e., their probabilistic predictions do not correspond to the empirical likelihood that the prediction is correct. Neural language models, such as, e.g., models for machine translation, risk to be miscalibrated, too (Kumar and Sarawagi, 2019). Various remedies for miscalibration have been proposed and explored in the literature: modification of the loss-function (Moon et al., 2020); coupling and training of a complementary network to predict a prediction's reliability, that is, its empirical likelihood of being correct (Corbière et al., 2019); simultaneous training of an entire ensemble of deep neural networks (Lakshminarayanan et al., 2017); model distillation (Guo H. et al., 2021).

Some pre-trained Transformers have, however, been claimed to be reasonably well calibrated. Thus, Radford et al. (2019) report that GPT-2's conditionally generated answers in a QA task are well calibrated. Similarly, Desai and Durrett (2020) find that fine-tuned BERT (Devlin et al., 2019) and RoBERTa (Liu et al., 2019) generate reliable probabilistic predictions across different NLU tasks—both in- and out-of-domain. Hendrycks et al. (2020), in contrast, evaluating GPT-3 with multi-disciplinary QA tasks, argue that the zero-shot calibration of GPT-3 is extremely poor. Likewise, Guo H. et al. (2021) insist that pre-trained RoBERTa is poorly calibrated out-of-the-box, too. Shwartz and Choi (2020), analyzing mis-calibration in terms of deviation from reporting frequencies, argue that pre-trained language models are well calibrated for prevalent and recurring judgments in the training corpus, but exhibit systematic bias for rare judgments.

In line with this mixed picture, we find that a pre-trained language model's confidence in judgment *s* is closely tied to the relative frequency of *s* being considered as true according to training sources—if and only if the training corpus is balanced w.r.t. *s* (cf. Section 4.1). We go beyond the literature in proposing a self-training procedure that effectively reduces global biases (mis-confidence) in a model's belief system (cf. Section 4.3).

### 2.3. NLMs as reasoners

While the zero-shot reasoning ability of PTLMs is agreed to be limited (e.g. Brown et al., 2020; Zhou et al., 2020), NLMs have been fine-tuned to reliably carry out formal deduction (Weber et al., 2019; Minervini et al., 2020) and natural-language inference (Banerjee et al., 2020; Clark et al., 2020; Betz et al., 2021b; Saeed et al., 2021). Moreover, NLMs have been successfully trained to generate natural language proof chains or multi-hop derivations of a conclusion from a given set of sentences, as demonstrated by ProofWriter (Tafjord et al., 2020), PRover (Saha et al., 2020), multiPRover (Saha et al., 2020), EntailmentWriter (Dalvi et al., 2021), Parapattern-BART (Bostrom et al., 2021), or the Transformer trained on CLUTRR data (Sinha et al., 2019) by Gontier et al. (2020).

This study parallels these proof-generating systems inasmuch as our pre-trained model is used to generate inferentially structured texts as well. However, unlike in the systems mentioned above, text generation during self-training is open-ended rather than goal-oriented (i.e., does not aim to proof a given conclusion); in addition, we effectively employ such generated argumentative texts to further self-train the model.

### 2.4. Self-training and self-improving NLMs

The learned skills of a NLM can be deployed for self-improvement both during inference and training. On the one hand, dynamic context expansion, i.e., the augmentation and/or modification of a task's input by the NLM at inference time, has been extensively studied in the context of commonsense QA (e.g., Chen et al., 2019; Lewis et al., 2020; Petroni et al., 2020; Shwartz et al., 2020) and reasoning (e.g., Saha et al., 2020; Betz et al., 2021a). On the other hand, semi-supervised learning, i.e., the automatic augmentation of unlabeled training data, is a widely implemented technique for self-training, which typically distinguishes a teacher-model for data augmentation, and a student-model being trained (Du et al., 2021; Mi et al., 2021; Seo et al., 2021). Yang et al. (2020) push the idea of self-training further by labeling *synthetic* examples that have been generated by a NLM. In a refinement of this approach, He et al. (2021) show that such self-training yields substantial improvements in commonsense reasoning and NLI performance.

In agreement with this literature, we train our models on self-generated texts during self-training. However, we deviate from the prevailing teacher-student paradigm: Teacher (generating training text) and student (being trained) are one and the same model. In consequence, text generation is dynamic and may adapt during the self-training processes (e.g., texts with different properties may be produced at the beginning of self-training as compared to at the end, see also Section 4.2). In these regards, our self-training procedure resembles iterative back-translation, which has been shown to improve the quality of machine translation, especially through correcting errors in the original training data (Guo Y. et al., 2021).

## 3. Technical design

The introduction has provided an informal overview of our computational experiments and motivated our general approach. In this section, we shall describe the methodological set-up more thoroughly. Section 3.1 explains how we generate synthetic training corpora by simulating groups of authors who hold beliefs about how to rank objects in a domain, and who generate texts by expressing those beliefs. It also introduces the artificial language used throughout the experiments. The two training phases (pre-training and self-training) are described in Sections 3.2, 3.3. Section 3.4 details the mask-prediction task we employ to elicit a model's beliefs. And Section 3.5 introduces the “doxastic metrics” for assessing the models' belief systems (e.g., with respect to consistency). Further technical details may be found in the Appendix (Supplementary material) and will be pointed to where appropriate.

### 3.1. Artificial corpus construction

We use a simple **artificial language** *L*—actually, a small fragment of 1st-order logic—to carry out our study. The language is designed so that it contains minimally inconsistent subsets of size 3, can be easily and unambiguously tokenized, and possesses a simple semantics.

The alphabet of *L* consists of 200 constants *a*_1_…*a*_200_ and two binary predicate letters *R, S*. All sentences in *L* are atomic, and have hence the form *xRy* or *xSy* (we use *x, y, z* as metavariables ranging over *L*'s constants). The logic of *L* is defined by the following four inference-rules (which are not expressible in *L* itself): irreflexivity (*xRx* ⇒ ⊥, for any *x*); asymmetry (*xRy, yRx* ⇒ ⊥, for any *x, y*); transitivity (*xRy, yRz* ⇒ *xRz*, for any *x, y, z*); duality (*xRy*⇔*ySx*, for any *x, y*).

Note that, because of duality, there exists, for every *L*-sentence, precisely one other logically equivalent *L*-sentence. For example, sentence *a*_3_*Sa*_2_ is equivalent to sentence *a*_2_*Ra*_3_.

And because of asymmetry, there exist, for every *L*-sentence, exactly two different logically contradictory *L*-sentences. Sentence *a*_3_*Sa*_2_, for example, is contradictory to *a*_2_*Sa*_3_, and to *a*_3_*Ra*_2_.

We say that *xRy* is the negation of *xSy*, and vice versa, and write $\bar{s}$ for the negation of sentence *s*.

The language *L* has a simple, natural **semantics**. A theory (set of sentences) in *L* is consistent (⊥ cannot be derived with the inference-rules) if and only if it can be interpreted as a strict order over a domain *D* of 200 items. Let us flesh out the semantics of *L* with a concrete model and consider the top-200 tennis players, 1, 2…200, as our domain *D*. Every constant in *L* is a unique name of one of these tennis players, and we may assume that *a*_1_ refers to player 1, *a*_2_ to player 2 etc. We interpret the binary relation *R* as expressing that one player is strictly taller than another player. This relation is irreflexive (no player is strictly taller than herself), asymmetric (if *i* is strictly taller than *j*, *j* cannot be strictly taller than *i*), transitive (if *i* is strictly taller than *j* and *j* is strictly taller than *k*, *i* is strictly taller than *k*), and hence matches the logic of *L*. Correspondingly, *S* stands for the relation that one player is strictly smaller than another. Under the assumption that no two players are of exactly the same height, both relations satisfy duality. In this interpretation, the sentence *a*_2_*Sa*_3_, e.g., means that player 2 is strictly smaller than player 3. We will resort to the tennis model of *L* to illuminate the further exposition of the technical framework; yet, note that it serves merely illustrative purposes and represents just one possible interpretation of the artificial language used in this study.

To generate text corpora in *L*, we simulate text production processes. We define **authors** as formal agents who hold consistent beliefs (in *L*) and can produce texts which express those beliefs. To simplify the semantic representation of an author's beliefs, we additionally assume that her beliefs can be interpreted as a strict total order over a sub-domain of *D*. In terms of the tennis model: An author sorts a subset of players by height, such as for example by means of the following descending ranking of the top-10 players except number 3 (sub-domain),


$$2,4,10,1,7,8,9,6,5.\text{ (}H^{*})$$

Now, the corresponding pairwise height comparisons represent all her beliefs, e.g., she believes that *a*_2_*Ra*_1_ and that *a*_9_*Sa*_1_ are true, she believes that *a*_2_*Sa*_4_ is false, and she *suspends judgment* vis-à-vis *a*_2_*Ra*_24_ and *a*_103_*Sa*_57_ (i.e., neither considers these sentences true nor false). Hence, an author's belief system (*B*) is a consistent *and closed* set of *L*-sentences. For example, because the author believes *a*_2_*Ra*_1_ and *a*_9_*Sa*_1_, she also believes the logical consequence *a*_2_*Ra*_9_.

We further distinguish two types of authors by means of a “reach** threshold**”: those who can express every *L*-sentence they believe (reach = ∞) when producing a text; vs. those who can only express a sentence of the form *xRy* or *xSy* if, loosely speaking, the rank-order difference between *x* and *y* according to their belief system lies below a given threshold reach (with reach < 200). For example, in a belief system corresponding to (H^*^) above, the rank-order difference between players 1 and 7 equals 1, whereas the rank-order difference between players 1 and 6 is 4. With unlimited reach threshold, an author holding that belief system can express both her beliefs that *a*_1_*Ra*_7_ and that *a*_1_*Ra*_6_; with reach = 3, however, she can only express the former, not the latter. The introduction of a reach threshold has the effect that an author's set of *expressible* beliefs is not necessarily deductively closed.

Our simulated authors randomly produce finite, truthful, unbiased, inferentially structured *L*-texts, i.e., sequences of expressible *L*-sentences *s*_1_, *s*_2_, …, *s*_*l*_. Texts are truthful because they only contain sentences the author believes to be true (*s*_*i*_ ∈ *B* for *i* = 1…*l*). Texts are unbiased because all of an author's expressible beliefs are equally likely to figure in a text by the author. Texts are inferentially structured because, rather than expressing an author's beliefs in random order, texts follow the logical implications defined by the inference-rules, in particular, they contain transitivity arguments (e.g., *xRy*, *yRz*, *xRz*) and duality arguments (*xRy*, *ySx*) as sub-sequences. Consider, for illustration, the following two texts:


$$\begin{array}{cc} {\text{text}}_{1}: & a_{6}Sa_{8}\;a_{2}Ra_{1}\;a_{9}Sa_{1}\;a_{2}Ra_{9},\\ {\text{text}}_{2}: & a_{11}Sa_{8}\;a_{2}Ra_{1}\;a_{9}Sa_{1}\;a_{2}Ra_{9}. \end{array}$$

Both are inferentially structured: the final sentence follows from the two preceding ones. An author who holds beliefs corresponding to (H^*^) may produce text_1_, provided her reach threshold is greater than 6. She cannot, however, produce text_2_, as the author does not believe that the first sentence in text_2_ is true (she is suspending judgement), and text_2_ is therefore not truthful to her beliefs. Appendix A gives further details of how authors sample texts.

We define a **society** as a group of *n* authors with belief systems *B*_*i*_ (*i* = 1…*n*) that share a specific set of background beliefs and produce, independently of each other, texts that collectively make up a **corpus**. A society's shared background beliefs, *K*, are modeled as a strict total order on subdomain *D*_*K*_ ⊂ *D*; every author's belief system then extends this shared order, *K* ⊂ *B*_*i*_ with *D*_*K*_ ⊂ *D*_*i*_ ⊆ *D* for *i* = 1…*n*. Let us assume, returning to the tennis model, that it is common knowledge how to rank players 4, 5 and 6 (*D*_*K*_ = {4, 5, 6}) in terms of height, namely as


$$\begin{array}{l} 4,6,5. \end{array}\text{ (}H^{K}\text{)}$$

The illustrative belief system represented by (H^*^) above shares and extends the background knowledge (*H*^*K*^).

We may characterize societies in terms of (i) the number of authors, (ii) the extent of shared background beliefs, as measured by the ratio |*D*_*K*_|/|*D*|, and (iii) the reach threshold which controls which beliefs authors can express in their texts. So as to cover, in our simulation study, a wide spectrum of boundary conditions, we define 3 × 4 × 2 corresponding profiles with

- n_authors = 5, 15, 25;
- background_ratio = 0, 0.25, 0.5, 0.75;
- reach = ∞, 50.

Note that in societies with reach = 50, authors can express less than half of the beliefs they may hold; and, importantly, the text corpus they produce is *not inferentially closed*. Put differently, such authors communicate efficiently: while they explicitly express less than half of their beliefs, everything they do believe *can be inferred* from what they (may) express.

For each of the 24 profiles, we create five different societies (by sampling shared background beliefs and the authors' belief systems), each of which collectively produces (with equal contributions by all authors) a corpus of 101,000 texts. This gives us 120 different societies and an equal number of corresponding text corpora.

As an additional characterization of a society's *diversity*, we measure the rank correlation between the strict orders which model the authors' belief systems. In particular, we resort to Kendall's tau correlation measure, finding that kendalltau varies between −0.03 and 0.22, with median value at 0.055. We split simulated societies in two equally sized groups, classified as exhibiting high diversity (kendalltau < 0.055) vs. high agreement (kendalltau> 0.055).

### 3.2. Pre-training regime

We train randomly initialized T5 models (Raffel et al., 2020; Wolf et al., 2020) on each society's text corpus with an equal share of masked language modeling (denoising) and text generation training items, which gives us in total 120 pre-trained models. We construct *denoising training items* by masking sequences in the raw training texts (in close analogy to the original pre-training regime of T5); moreover, *text generation training items* consist in an initial sub-sequence of a given text (as input) and the full text (as target). Models are accordingly trained on a given corpus (which is randomly divided into a *train* split containing 100,000 raw texts and an *eval* split with 1,000 texts) for 18 epochs or until *eval loss* doesn't decrease any further. Appendix C provides further technical details.

We have chosen masked token prediction and linear text completion as pre-training tasks because our belief elicitation procedure (cf. Section 3.4) is based on masked token prediction, and the self-training regime (cf. Section 3.3) requires that models are able to generate texts. This dual demand has also guided our choice of transformer architecture (seq-to-seq rather than decoder- or encoder-only models)—whereas the experiments presented here could in principle be carried out with causal LMs, too, by adapting the belief elicitation procedure.

### 3.3. Self-training regime

Every pre-trained model is submitted to four independent self-training runs, which consist in 600 training steps. At each step in a self-training loop, the model generates texts, which are processed, filtered, masked, and finally used as training data for denoising training (see Appendix D, Algorithm 2). More specifically, we generate, first of all, 200 prompts by sampling strong beliefs from the model (see also Appendix D, Algorithm 3). Being queried with each of these prompts, the model returns, with beam sampling, 5 generated text sequences and corresponding scores. Texts are split into sub-sequences of length 3, discarding all sub-sequences which do not represent a syntactically well-formed sentence. Next, we keep only sentences from texts with at least 6 well-formed sentences and high beam scores (top 15%). These sentences are transformed into training data by masking their predicate letters—similarly to the masking for belief elicitation (cf. Section 3.4). Finally, the model is trained on a denoising task with the thusly generated training items for one epoch.

We define a simple **baseline** in close correspondence to self-training by drawing texts from the original corpus rather than letting the model generate raw training texts itself.

### 3.4. Belief elicitation and sentence-wise vote ratios

Let *M* be a neural language model capable of masked token prediction in our language *L*. To elicit the model's belief in a *L*-sentence *a*_*i*_*Ra*_*j*_ (likewise *a*_*i*_*Sa*_*j*_), we mask the predicate letter, *a*_*i*_[mask]*a*_*j*_, query the model, and interpret the model's probability prediction for [mask] = *R*, the so-called confidence, as its degree of belief in *a*_*i*_*Ra*_*j*_, in short:


$$\begin{array}{l} {\text{BEL}}_{M}\left(a_{i}Ra_{j}\right)=\text{Pro}{\text{b}}_{M}\left(\;\text{[mask]}=R|a_{i}\;\text{[mask] }a_{j}\right). \end{array}$$

Since we will compare a model's degrees of belief with the authors' beliefs in a society, we introduce the sentence-wise vote ratio as a simple belief aggregation method. Consider a society containing *n* authors with belief systems *B*_*i*_ (*i* = 1…*n*) and shared reach threshold *r*. Let $B_{i}^{r}$ denote the corresponding set of *expressible* beliefs of author *i*. We define the society's sentence-wise vote ratio in the *L*-sentence *s* as


$$\text{VR}(s)=\frac{1}{n}\sum_{i=1}^{n}v(i)\text{ with }v(i)=\{\begin{array}{ll} 1 & \text{if }s\in B_{i}^{r}\\ 0 & \text{if }\bar{s}\in B_{i}^{r}\\ 0.5 & \text{otherwise} \end{array}\;.$$

The sentence-wise vote ratio generalizes binary sentence-wise majority voting.

### 3.5. Doxastic metrics

The following metrics can be applied both to degrees of belief elicited from a model and, likewise, to vote ratios aggregated from belief systems of authors. To keep the presentation plain, we shall introduce them, below, as doxastic metrics only.

First of all, **transitivity violation** is one reason for why degrees of belief may be logically incoherent. Let *s*_1_, *s*_2_, *s*_3_ be three minimally inconsistent *L*-sentences (such as, e.g., *a*_1_*Ra*_2_, *a*_2_*Ra*_3_, *a*_1_*Sa*_3_), i.e., any two of these statements imply, with transitivity (and, possibly, duality), the negation of the remaining one. Now, let *x*_1_, *x*_2_, *x*_3_ be degrees of belief assigned to these three statements [*x*_*i*_ = BEL(*s*_*i*_)]. For definiteness, we may assume *x*_1_ ≤ *x*_2_ ≤ *x*_3_. Informally speaking, the degrees of belief violate the transitivity rule in case *x*_1_, *x*_2_, *x*_3_ are all too high (at least one statement has to be dis-believed). In particular, as *s*_2_ and *s*_3_ jointly imply the negation of *s*_1_, either the conjunction of *s*_2_ and *s*_3_, or *s*_1_ must not be believed. We may resort to fuzzy logic (see Appendix B) to spell out this constraint as a precise inequality,


$$\begin{array}{l} x_{1}+x_{2}-1\leq 0. \end{array}\text{ (TC)}$$

We will say that *x*_1_, *x*_2_, *x*_3_ violate the transitivity constraint iff they violate the above inequality (3), in which case *x*_1_+*x*_2_−1 expresses the *degree of transitivity violation*. Let us suppose, for example, that *x*_1_ = 0.4, *x*_2_ = 0.5, and *x*_3_ = 0.8. In this case, the transitivity constraint is not violated, as *x*_1_ and *x*_2_ add up to 0.9 ≤ 1. Consider, in contrast, slightly higher degrees of belief *x*_1_ = 0.5, *x*_2_ = 0.7, *x*_3_ = 0.8: this belief profile violates the transitivity constraint with degree 0.2. Note that it is only by considering the *degree of transitivity violation* (in addition to observing whether TC is satisfied) that we may evaluate the latter case differently from a situation where all three collectively inconsistent statements are maximally believed (*x*_1_ = *x*_2_ = *x*_3_ = 1).

For a *set of* minimally inconsistent triples and corresponding degrees of belief, we may thus calculate (i) the *ratio of transitivity violations* and (ii) the *mean degree of transitivity violation*.

The degree of **informativeness** expresses how extreme—close to either 1 or 0—the beliefs in a system are. We use normalized variance as a simple measure of informativeness (stipulating ${\text{BEL}}_{M}\left(s\right)+{\text{BEL}}_{M}\left(\bar{s}\right)=1$ and hence μ = 0.5). More precisely, let *X* = 〈*x*_1_…*x*_*k*_〉 be some degrees of belief, then $\text{inf}\left(X\right)=\frac{1}{k}\overset{k}{\underset{i=1}{\sum }}{\left(1-2x_{i}\right)}^{2}$. This measure of informativeness is (for fixed *k*) negatively correlated with, and hence a proxy for, the joint entropy of the degrees of belief (assuming independence). We will assess **global over-** and **under-confidence** of a model's degrees of belief relative to a society's collective judgments in terms of a mismatch of informativeness. In particular, with *L*-sentences *s*_1_, …, *s*_*k*_, *X* = 〈BEL_*M*_(*s*_1_), …,BEL_*M*_(*s*_*k*_)〉 and *Y* = 〈VR(*s*_1_), …,VR(*s*_*k*_)〉, we say that model *M* is globally over-confident if inf(*X*) > inf(*Y*), and globally under-confident in the opposite case. Mis-confidence, accordingly defined, is a specific (namely, systematically biased) form of mis-calibration: confidence levels do not only deviate from empirical frequencies (vote ratios), but they do so in a biased way, e.g., systematically over-estimating the empirical frequencies.

Finally, we may want to measure the overall disagreement between two belief systems. Let *X* = 〈*x*_1_…*x*_*k*_〉, and *Y* = 〈*y*_1_…*y*_*k*_〉 be degrees of belief assigned to *L*-sentences *s*_1_…*s*_*k*_. We may now use the relative entropy (Kullback-Leibler divergence) as a measure for how much *X* diverges from *Y*, $\text{KL}\left(X||Y\right)=\overset{k}{\underset{i=1}{\sum }}x_{i}\log\left(x_{i}/y_{i}\right)$. We will estimate the **volatility** of consecutive belief system changes by tracking KL(*X*_*t*+1_||*X*_*t*_), and we will measure the **global divergence** of an evolving belief system at step *t* from a given initial state by KL(*X*_*t*_||*X*_0_).

Each doxastic metric introduced in this section is calculated for a given set of *L*-sentences (or, in the case of transitivity violation, a set of inconsistent *L*-triples). It is, however, impractical to compute these metrics for *all* *L*-sentences in the experiments reported below. Therefore, whenever we determine a doxastic metric, we do so for a random sample containing 1,000 *L*-sentences, which are drawn independently of each other (and irrespectively of the agents' reach thresholds) by randomly choosing (i) two different constants (*a*_*i*_, *a*_*j*_ with 1 ≤ *i*≠*j* ≤ 200) and (ii) a binary relation *R* or *S*.

## 4. Results

### 4.1. Neural belief formation as judgment aggregation

Do pre-trained models aggregate a society's judgments sentence-wise? To answer this question, we elicit a model's degrees of belief for a random sample of sentences *S* and compare those with the society's corresponding vote ratios. Table 1 reports the thusly calculated mean squared deviation, distinguishing—per column:—between sentences according to the proportion of authors who suspend judgment with respect to the sentence, and aggregating—per row:—over all societies with the same number of authors and reach threshold. Formally, let *M*_1_, …, *M*_*k*_ be all models trained on a society with a given number of authors and reach threshold, let *S*_1_, …, *S*_*k*_ be random samples of *L*-sentences, and let *R* be a given real interval (bin). We write $S_{i}^{R}\subseteq S_{i}$ for the set of sentences *s* such that the ratio of authors in the underlying society *i* who (i) hold a belief about *s* and (ii) can express *s* given their reach threshold lies within *R*. Table 1 displays $\frac{1}{k}\overset{k}{\underset{i=1}{\sum }}\frac{1}{\left|S_{i}^{R}\right|}\underset{s\in S_{i}^{R}}{\sum }{\left({\text{BEL}}_{M_{i}}\left(s\right)-{\text{VR}}_{i}\left(s\right)\right)}^{2}$.

**Table 1:** Mean squared deviation (MSD) between a model's degrees of belief and the underlying society's sentence-wise vote ratios.

		**Proportion of authors holding a belief about and being able to express the sentence (bin)**			
		**[0, 0]**	**(0, 1/3]**	**(1/3, 2/3]**	**(2/3, 1]**
**Reach**	**Authors**				
∞	5	0.179	0.106	0.086	0.024
	15	0.117	0.097	0.071	0.022
	25	–	0.055	0.038	0.018
50.0	5	0.197	0.162	0.122	0.017
	15	0.156	0.129	0.076	0.014
	25	0.156	0.083	0.067	0.010

Sentences are classified (columns) according to the proportion of authors in a society that hold and can express a belief about the sentence, i.e., the MSDs are reported for different levels of judgment suspension (with decreasing rate of judgment suspension from left to right). MSDs are, moreover, averaged over sentences from societies with a given number of authors and reach threshold (rows). MSD values are omitted (–) if the corresponding sub-sample size is very small.

The main take-away is that the difference between a society's vote ratio for some sentence *s* and a model's corresponding degree of belief is small provided that most authors hold an expressible belief about *s* (right column in Table 1). For higher ratios of judgment suspension, we observe substantially greater differences, especially in societies with few authors or limited reach threshold. In other words, if a training corpus is biased (e.g., a sentence *s* is underrepresented), a model's degrees of belief may diverge from sentence-wise vote ratios. However, we find the best match between degrees of belief and vote ratios in case of low judgment suspension *and* limited reach threshold (right-most column, lines 3–6). That is because, in these cases, the authors communicate efficiently (cf. Section 3.1): there is a lower number of different statements they express in texts, but (given same corpus size) each statement they do express will be uttered more frequently, both in absolute and relative terms. Specifically, a statement *s* about which nearly all authors express their belief (as either *s* or $\bar{s}$) will occur *twice as frequently* in the entire corpus if reach = 50 as compared to reach = ∞. This increased presence in the training corpus may explain the closer match between degrees of belief and vote ratios.

**Figure 2:**
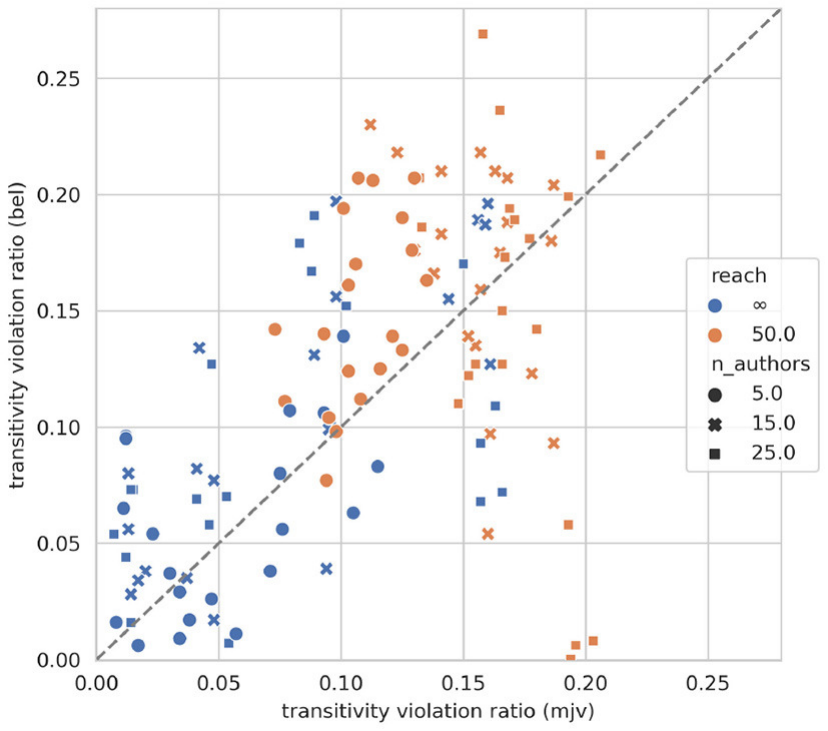
Initial transitivity violation according to degrees of belief (*y*-axis) and sentence-wise vote ratios (*x*-axis).

Social choice theory implies, as noted above, that sentence-wise aggregation *can* result in logical inconsistencies. To which extent does this actually happen in our pre-trained models? Figure 2 displays the relative frequency of transitivity violations (see Section 3.5) for all societies and corresponding pre-trained models according to vote ratios (*x*-axis) and degrees of belief (*y*-axis). We observe that, first, the ratio of inconsistencies spreads widely and may be substantial, with some models violating as many as 1 out of 5 transitivity constraints. Second, doxastic transitivity violation (by a model) correlates clearly with vote ratio transitivity violation (Pearson's *r* = 0.54). This strongly suggests, for lack of an alternative explanation, that the observed incoherence of models' degrees of belief is actually due to their particular sentence-wise judgment aggregation. The models run into discursive dilemmas because they form beliefs, during pre-training, in accordance with sentence-wise vote ratios.

### 4.2. Coherence increase through self-training

**Figure 3:**
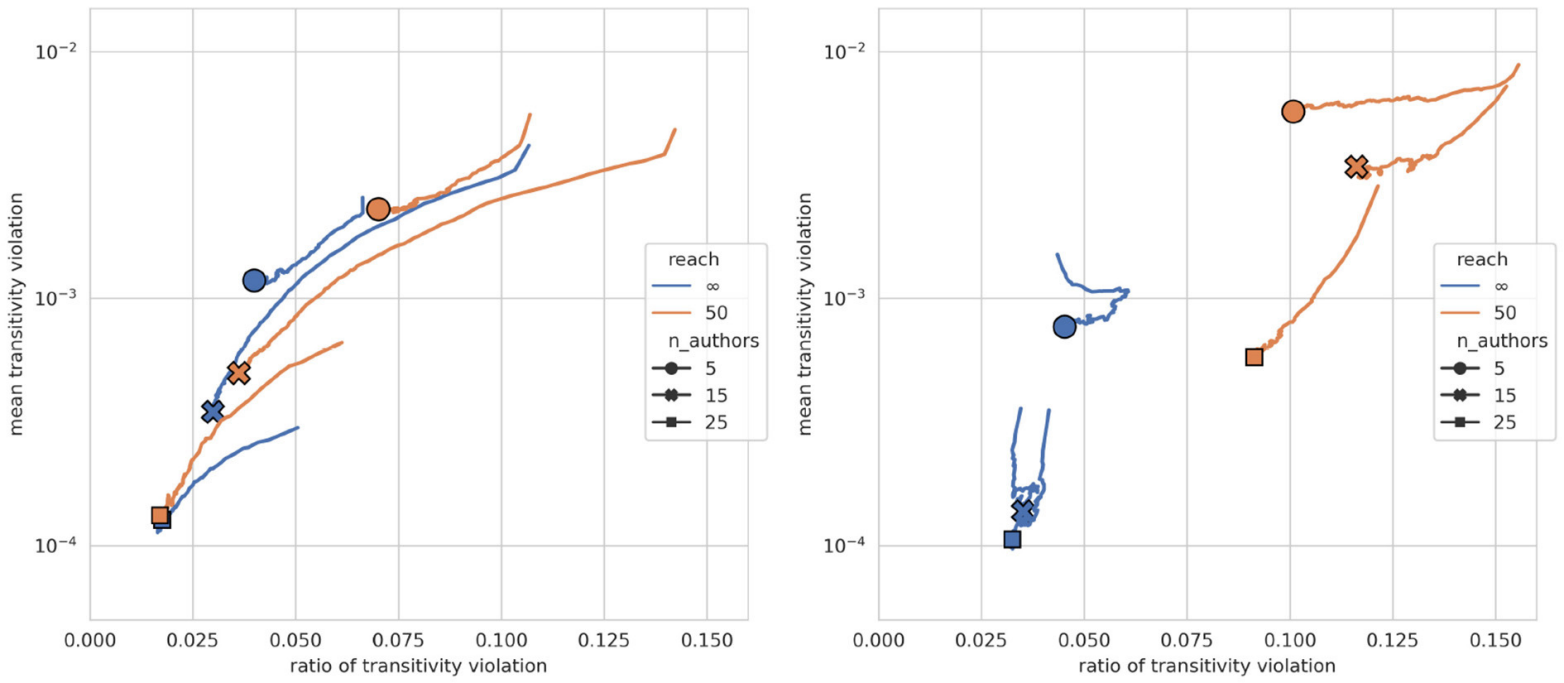
Transitivity violation trajectories during self-training. Markers indicate the final state reached after self-training. **Left**: models pre-trained on a corpus with high inter-author diversity (kendall_tau < 0.055); **right**: models pre-trained on a corpus with low inter-author diversity (kendall_tau > 0.055). A trajectory averages over all models pre-trained on a corpus with a given number of agents (indicated by marker style) and a given reach threshold (indicated by color).

What is the effect of self-training on the level of incoherence of a model's beliefs? Figure 3 plots the models' trajectories during self-training in a logical phase space—i.e., frequency of transitivity violations on the *x*-axis, mean degree of transitivity violations on the *y*-axis—, summarizing the time-series shown in Figures E.3, E.4 in the Appendix. It aggregates evolutions of models pre-trained on societies with the same number of authors, same reach threshold, and similar inter-author agreement. We see, first and foremost, that self-training drastically reduces incoherence: models move, along the trajectories, toward the plots' origins. For example, in societies with 15 authors, high diversity and limited reach (left-hand plot, orange trajectory marked with cross), the frequency of transitivity violations (*x*-axis) is brought down from roughly 15% to less than 4% with a simultaneous reduction in the mean degree of violation (*y*-axis). *Ceteris paribus*, these logical improvements of the models' belief systems (which show in the direction and length of the trajectories) are more substantial in societies with high divergence, for corpora that are not inferentially closed (i.e., reach = 50), and for models with high initial levels of incoherence. Consider, for example, societies with 25 authors (trajectories marked with a square) and compare models pre-trained on high-diversity corpora (left-hand plot) vs. those pre-trained on doxastically homogeneous, high-agreement corpora (right-hand plot): Models that have been exposed to highly diverse texts during pre-training (left-hand plot) do not only exhibit greater improvement *relative* to the initial level of inconsistency, but eventually even attain, *in absolute terms*, a lower ratio of transitivity violation.

In the fine-tuning **baseline**, where models further train on texts from the corpus rather than on self-generated ones, there is no comparable improvement of beliefs, and the levels of incoherence stay generally far above those observed during self-training (cf. Figures E.3, E.4 in Appendix).

Why does self-training improve the models' beliefs? To better understand how a model modifies its beliefs, and for such diagnostic purposes only, we're parsing and logically evaluating the self-generated texts. This reveals, first, that the texts are, *cum grano salis*, inferentially structured and coherent. More precisely, while most sentences are logically independent (neutral) of the sentences previously stated in a text, the ratio of sentences that follow from what has been previously asserted is far greater than the ratio of contradictions (cf. Figure E.5 in Appendix). Moreover, belief elicitation reveals that the model occasionally assigns low degrees of belief to sentences in its self-generated texts (cf. Figure E.6 in Appendix). Or, put differently, the model asserts sentences in its texts which it actually disbelieves. All this points toward a mechanism of rational belief revision: In composing an inferentially structured text, starting with its own beliefs and drawing conclusions from what has been written before, the model locally spells out consequences of its beliefs and is brought—by the “unforced force” (Habermas, 1996) of valid inference—to assert sentences it may actually disbelieve. Training on these sentences then triggers a corresponding, coherence-conducive belief revision.

### 4.3. Mis-confidence correction through self-training

**Figure 4:**
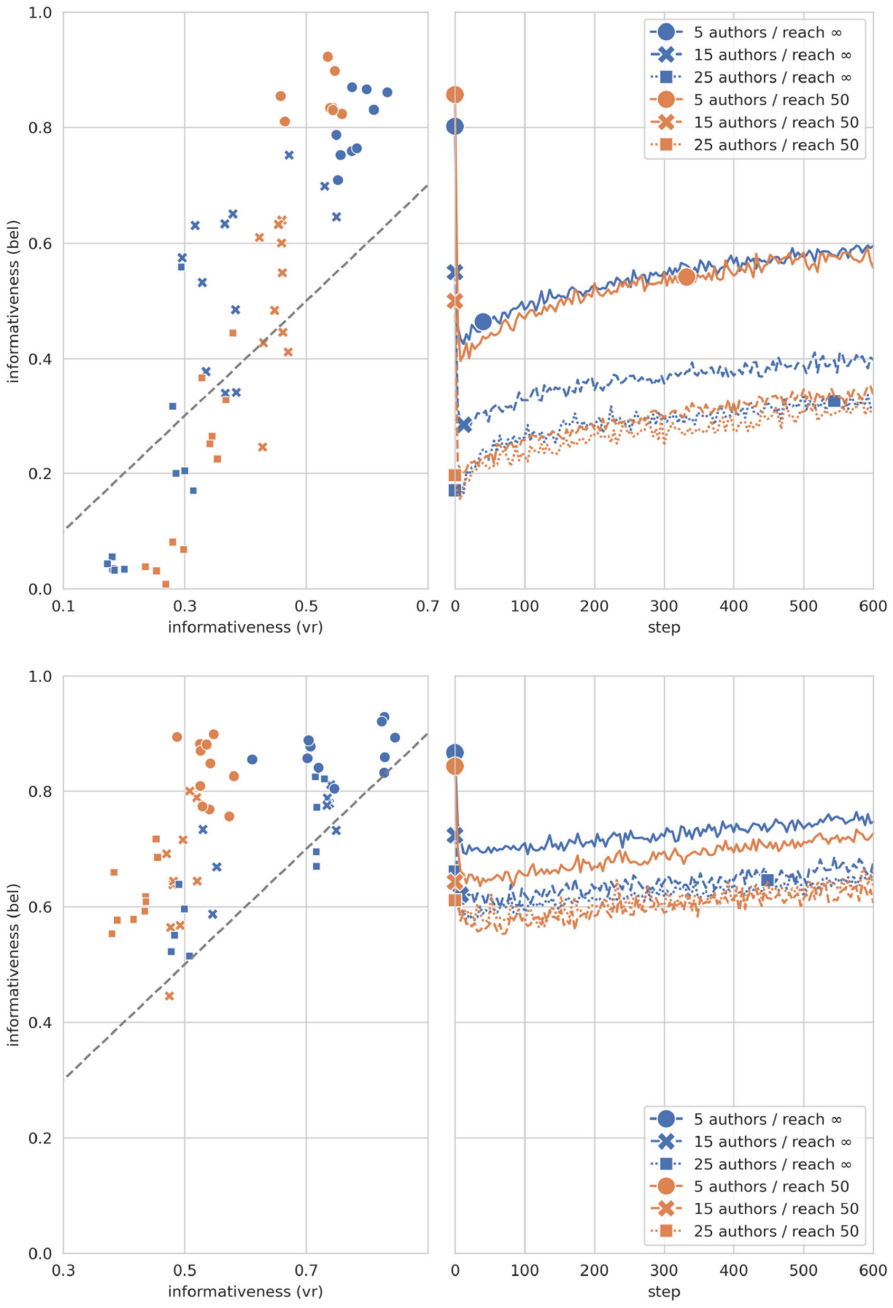
Initial over- and under-confidence (scatter-plots), and informativeness evolution during self-training (line-plots). **Top**: corpora with high inter-author diversity (kendall_tau < 0.055); **bottom**: corpora with low inter-author diversity (kendall_tau > 0.055). In the *scatter-plots*, each marker represents a single pre-trained model, which exhibits over-confidence (under-confidence) if and only if its marker lies well above (below) the dotted diagonal. In the *line-plots*, the shown trajectories average over all models with the same number of agents and same reach threshold; the first marker designates the mean initial doxastic informativeness, the second one marks the step from which on mean doxastic informativeness is greater than average initial vote ratio informativeness.

Are pre-trained models initially over- (or under-) confident, and how does self-training affect such mis-confidence? As the scatter-plots in Figure 4 show, pre-trained models tend to be globally over-confident (in the sense of Section 3.5): their degrees of belief are more informative than the collective vote ratios of the corresponding authors. As an exception, models trained on societies with many, strongly disagreeing authors are under-confident. Now, self-training corrects such mis-confidence in characteristic ways, as shown by the line-plots in Figure 4. In cases of initial under-confidence, self-training gradually increases the informativeness of the models' beliefs. In cases of over-confidence, self-training decreases informativeness immediately and sharply. This initial (typically over-shooting) correction tends to result in a state of under-confidence. Further self-training then gradually increases informativeness—in some cases even over and above the informativeness of collective vote ratios (as indicated by a second marker on a curve). We suggest that such a final surplus of informativeness is not necessarily a sign of global mis-confidence (i.e., error), but may simply reflect that the model has rationally consolidated its belief system. After reflective equilibration, the model may well be justified in holding beliefs that are more informative than the original collective vote ratios. All in all, self-training modifies the informativeness of a model's beliefs in—it seems—appropriate and reasonable ways.

### 4.4. Convergence during self-training

Does a model's belief system converge during self-training? In each self-training run, we are tracking the beliefs of the model on a fixed random sample of *L*-sentence, which allows us to estimate the extent of gradual belief change (volatility) and the global divergence of the belief system from the initial belief state. The most extensive belief change, we observe, takes place at the beginning of self-training, volatility then drops quickly, and further decays continuously (cf. Figure E.7, top row in Appendix). Likewise, the belief systems quickly diverge from the initial state, after which global divergence increases less and less slowly and eventually settles, or so it seems, at some level (cf. Figure E.7, bottom row in Appendix). Both volatility and global divergence from the initial state are more pronounced for models trained on high-diversity corpora than those trained on low-diversity corpora. In sum, we find that belief systems are not only improved during self-training, but tend to *converge* to a new belief state.

Further evidence for the seeming convergence of a model's belief system during self-training is provided by the observation that the model's degrees of belief in the most dis-believed sentences of its self-generated texts substantially increase during self-training (cf. Figure E.6 in Appendix). More and more, the model reaches a point where it believes what it says (or writes). This increasing confidence in the self-generated texts means that, as training data, these texts will trigger ever smaller belief revisions.

## 5. Conclusion

Social choice theory may help us to understand why the output of neural language models is frequently inconsistent. We show, in a fully synthetic experimental set-up, that NLMs aggregate judgments according to sentence-wise vote ratios, which inevitably leads to so-called discursive dilemmas (cf. Section 4.1). In particular, we diagnose that a pre-trained model's beliefs are *ceteris paribus* more incoherent if the training corpus is highly diverse or not inferentially closed. As a remedy, we propose a self-training procedure—inspired by the method of reflective equilibrium—that effectively reduces the extent of logical incoherence in a model's belief system (cf. Section 4.2), corrects global mis-confidence (cf. Section 4.3), and eventually allows the model to settle on a new belief state (cf. Section 4.4). The logical improvements induced by self-training are especially pronounced if the initial beliefs are extremely inconsistent; and it's precisely in these cases where we observe the furthest deviations of a model's belief system from the initial state during self-training. Moreover, inconsistencies are not simply resolved by giving up more and more beliefs: On the contrary, the continuous coherence increase during self-training goes hand in hand with a simultaneous growth of informativeness.

Training on self-generated texts is not only instrumentally rational (in bringing about doxastic improvements), but seems to be driven by a mechanism of reasonable belief revision, as additional diagnostic evidence suggests. Specifically, we find that self-generated texts are inferentially structured and can hence be considered to locally spell out logical consequences of a model's beliefs. But as the model, occasionally, strongly disbelieves some of these consequences, training on self-generated texts leads to a gradual revision of the corresponding beliefs. Conceptually, the more a text is disbelieved, the stronger a belief revision it induces. If, conversely, a model's texts express more or less exactly what the model believes, text production and belief system are in sync and the model has reached an equilibrium belief state that is not revised any further. Accordingly, we observe that models which undergo the most far-reaching belief revisions (in terms of coherence improvement and global deviation from the initial state) most strongly doubt—at least initially—the sentences in their self-generated texts. Also, this rational revision mechanism may explain why models pre-trained on highly diverse text corpora initially suffer from wide-spread inconsistencies, but are able to considerably self-improve their belief state nonetheless. That is because what drives rational belief revision is the ability to spell out consequences of one's beliefs, i.e., to generate logically structured texts. Now, while corpus diversity obviously hampers the consistent memorization of facts, it still allows for, and possibly even facilitates the learning of inferential structures and the reproduction of argumentative patterns in texts.

So, for the self-training language model, logical coherence is an emergent phenomenon. Consistency is not built into our system as an explicit goal or constraint (unlike, e.g., in Kassner et al., 2021b). Accordingly, and pace theories of cognitive consistency (Festinger, 1964; Gawronski and Strack, 2012), consistency-conducive cognition does not *necessarily* require a corresponding psychological motivation (such as resolving emotional dissonance)—which is not to deny that a motive to resolve inconsistency, too, *can* trigger coherence-increasing changes in belief.

Our study is limited in various and obvious ways, some of which we shall highlight here.

**Training regimes**. We have set-up our particular pre-training regime in analogy to the original denoising training of T5 (Raffel et al., 2020); whereas the self-training design, inspired by the method of reflective equilibrium, has been informed by pre-studies without being systematically optimized. So, it is unclear whether variations of our self-training method give rise to different, stronger or weaker doxastic improvements. And it is equally unclear whether different pre-training tasks will exacerbate, or mitigate the emergence of logical incoherence in the first place.

**Artificial language**. Our simple artificial language is logically just rich enough to allow for discursive dilemmas. It is unclear how the findings would be affected if the corpora were composed of texts in a more complex language with much more syntactic diversity, e.g., a language with quantification, with complex sentences, or with modal operators. Such complications would also open up further possibilities for eliciting beliefs as well as for designing a self-training regime.

**Social dynamics**. Our models reflect and revise their belief states in isolation. What happens if the self-training models start to interact? We don't know, though the literature on the emergence of natural language in deep multi-agent systems (Lazaridou et al., 2017; Lazaridou and Baroni, 2020) suggests that adding social dynamics might have profound effects (e.g., meaning shifts, conceptual revisions) beyond mere inconsistency correction. There exist multiple kinds of interaction that could be investigated in this study's framework: Models could self-generate training texts in dialogues rather than monologically; models could train on texts generated by peers; and models could elicit each others' beliefs and assess mutual trustworthiness (cf. Zollman, 2013; Flache et al., 2017).

**Ground truth**. In the current experimental design, a corpus may be more or less diverse (reflecting the level of inter-author agreement), but there is no ground truth. However, such a ground truth may be easily introduced into the set-up. This would allow one to study (i) the models' ability to track the truth during pre- and self-training, and (ii) the extent to which this ability depends, e.g., on the accuracy or diversity of the underlying text corpus.

**Transfer to natural language**. To which degree do the clear results we obtain in our fully artificial set-up apply to NLMs trained on natural language data? Let us first note that our diagnosis and proposed remedy are consistent with previous findings on reporting frequencies (Shwartz and Choi, 2020), respectively self-improvement *via* iterative back-translation (Guo Y. et al., 2021). Nonetheless, we concede that this does not settle the transferability question. This paper's study merely *suggests* explanatory hypotheses. And it investigates specific mechanisms *in isolation*. To understand, e.g., whether the observed inconsistency of natural language NLMs is actually induced by discursive dilemmas, NLMs (of different architecture and size) and training datasets would have to be systematically probed in specific ways. Moreover, only experimental studies can reveal whether a self-training procedure, similar to the one described here, may help natural language NLMs to improve their belief state as well.

In sum, we submit that our study raises a variety of fruitful questions that may be pursued in future research. More generally, by demonstrating that NLMs' inconsistencies can be explained in terms of discursive dilemmas and may be resolved by reflective equilibration, it encourages the further exploration of philosophical concepts and theories in the domain of AI and NLP.

## Data availability statement

The original contributions presented in the study are included in the article/Supplementary material, further inquiries can be directed to the corresponding author.

## Author contributions

All authors listed have made a substantial, direct, and intellectual contribution to the work and approved it for publication.

## Funding

This work was supported by the Helmholtz Association Initiative and Networking Fund on the HAICORE@KIT partition.

## Conflict of interest

The authors declare that the research was conducted in the absence of any commercial or financial relationships that could be construed as a potential conflict of interest.

## Publisher's note

All claims expressed in this article are solely those of the authors and do not necessarily represent those of their affiliated organizations, or those of the publisher, the editors and the reviewers. Any product that may be evaluated in this article, or claim that may be made by its manufacturer, is not guaranteed or endorsed by the publisher.

## Footnotes

^1^Prompting https://huggingface.co/xlm-roberta-large (as of 11/23/2021) with “Warsaw lies < mask> of Berlin.” etc.

^2^Prompting https://delphi.allenai.org (as of 11/23/2021) with “Should I hurt the cat so that the dog can survive, which it couldn't otherwise?,” “Should I hurt the dog so that the cat can survive, which it couldn't otherwise?,” and “Should I let the cat and the dog die?”.
