# Supplementary material for: Judgment aggregation, discursive dilemma and reflective equilibrium: Neural language models as self-improving doxastic agents
Source: Front Artif Intell. 2022 Oct 18;5:900943. doi: 10.3389/frai.2022.900943 (PMC9623417; doi:10.3389/frai.2022.900943)
Supplement: Supplementary file 1 [file Data_Sheet_1.pdf]

APPENDIX to: Judgment Aggregation, Discursive Dilemma and Reflective Equilibrium: Neural Language Models as Self-Improving Doxastic Agents

Gregor Betz
Karlsruhe Institute of Technology
Karlsruhe, Germany
gregor.betz@kit.edu

Kyle Richardson
Allen Institute for AI
Seattle, WA, USA
kyler@allenai.org

Abstract

Neural language models (NLMs) are susceptible to producing inconsistent output. This paper proposes a new diagnosis as well as a novel remedy for NLMs' incoherence. We train NLMs on synthetic text corpora that are created by simulating text production in a society. For diagnostic purposes, we explicitly model the individual belief systems of artificial agents (authors) who produce corpus texts. NLMs, trained on those texts, can be shown to aggregate the judgments of individual authors during pre-training according to sentence-wise vote ratios (roughly, reporting frequencies), which inevitably leads to so-called discursive dilemmas [Pettit, 2001]: aggregate judgments are inconsistent even though all individual belief states are consistent. As a remedy for such inconsistencies, we develop a self-training procedure — inspired by the concept of reflective equilibrium [Rawls, 1971] — that effectively reduces the extent of logical incoherence in a model's belief system, corrects global mis-confidence, and eventually allows the model to settle on a new, epistemically superior belief state. Thus, social choice theory helps to understand why NLMs are prone to produce inconsistencies; epistemology suggests how to get rid of them.

References

Philip Pettit. Deliberative democracy and the discursive dilemma. *Philosophical Issues*, 11:268–299, 2001. ISSN 15336077, 17582237. URL http://www.jstor.org/stable/3050604.

Colin Raffel, Noam Shazeer, Adam Roberts, Katherine Lee, Sharan Narang, Michael Matena, Yanqi Zhou, Wei Li, and Peter J Liu. Exploring the limits of transfer learning with a unified text-to-text transformer. *Journal of Machine Learning Research*, 21:1–67, 2020.

John Rawls. *A Theory of Justice*. Harvard University Press, Cambridge, 1971.

Thomas Wolf, Lysandre Debut, Victor Sanh, Julien Chaumond, Clement Delangue, Anthony Moi, Pierric Cistac, Tim Rault, Rémi Louf, Morgan Funtowicz, Joe Davison, Sam Shleifer, Patrick von Platen, Clara Ma, Yacine Jernite, Julien Plu, Canwen Xu, Teven Le Scao, Sylvain Gugger, Mariama Drame, Quentin Lhoest, and Alexander M. Rush. Transformers: State-of-the-art natural language processing. In *Proceedings of the 2020 Conference on Empirical Methods in Natural Language Processing: System Demonstrations*, pages 38–45, Online, October 2020. Association for Computational Linguistics. URL https://www.aclweb.org/anthology/2020.emnlp-demos.6.

A Text Production by Simulated Authors

Algorithm 1: Text-Production by Simulated Author

```
Input: p_inf = .6; p_dual = .2; p_append = .95;
min_length // minimum length of text to be produced
theory // belief system of the author
reach // reach threshold of the author
Result: text
begin
    text = ← ∅ ;
    while length of text < min_length do
        if random() < p_inf then
            // Compose argument
            sequence ← sample transitivity inference with true sentences according to theory and
              within reach ;
            sequence ← randomly paraphrase sentences in sequence ;
            if random() < p_dual then
                sequence ← append paraphrase of conclusion to sequence
            end
        else
            // Compose fact
            sequence ← sample single true sentence according to theory and within reach ;
            sequence ← randomly paraphrase sequence ;
            if random() < p_dual then
                sequence ← append paraphrase of sequence to sequence
            end
        end
        if random() < p_append then
            // Append new sequence at end of text
            text ← text + sequence;
        else
            // Insert sequence earlier
            text1, text2 ← split text randomly into two lists of sentences ;
            text ← text1 + sequence + text2
        end
    end
end
```

B Transitivity Violation Constraint

Let s_1, s_2, s_3 be three minimally inconsistent L-sentences, i.e., any two statements imply, with transitivity, the negation of the remaining one. And let $x_i = \text{BEL}_M(s_i)$ for $i = 1 \ldots 3$. For definiteness, we may assume $x_1 \leq x_2 \leq x_3$. Because $s_2, s_3 \Rightarrow \bar{s_1}$, we have, by the definition of entailment in many-valued logic,

$$\text{BEL}_M(s_2 \& s_3) \leq \text{BEL}_M(\bar{s_1}).$$

where $\text{BEL}_M(s_2 \& s_3)$ may be defined in accordance with the Gödel t-norm of conjunction as

$$\text{BEL}_M(s_2 \& s_3) := \min(\text{BEL}_M(s_2), \text{BEL}_M(s_3)).$$

Since $\text{BEL}_M(s_2) \leq \text{BEL}_M(s_3)$ and assuming $\text{BEL}_M(\bar{s}) = 1 - \text{BEL}_M(s)$ for every L-sentence s, we find

$$\text{BEL}_M(s_2) = \min(\text{BEL}_M(s_2), \text{BEL}_M(s_3)) \leq \text{BEL}_M(\bar{s_1}) = 1 - \text{BEL}_M(s_1),$$

and thus

$$x_1 + x_2 - 1 \leq 0.$$

C Pre-Training

The entire experiment is technically implemented within the HuggingFace Transformers framework [Wolf et al., 2020].

Given a text corpus, a randomly initialized T5 model (default configuration of Wolf et al. [2020], similar to T5-small) is pre-trained on two tasks: a denoising task and a text-completion task.

The *denoising task* is designed in analogy to pre-training of T5 [Raffel et al., 2020]. A raw text T from the corpus is transformed into a text2text training item as follows: First, a random sample of tokens from T is masked (mask probability equals 0.15, and the maximum length of a masked sub-sequence is 2). The masked text (with special mask tokens) serves as input sequence. The corresponding target sequence resolves the masks.

The *text completion task* splits a raw text T after some randomly chosen sentence into two sub-sequences T_1, T_2 with at least one sentence each. T_1 serves as input sequence of the training item, and the complete text T_1T_2 as its target sequence.

Each raw text in the training corpus yields both a denoising task item and a text completion task item, which jointly make up the pre-training dataset.

The randomly initialized models are pretrained (adafactor optimizer, 20.000 warmup steps, per device train batch size = 64) for 18 epochs with early stopping (given eval loss).

We observe, as shown in Figure C.1, characteristic training progress, with eval loss dropping markedly around epoch 6–10.

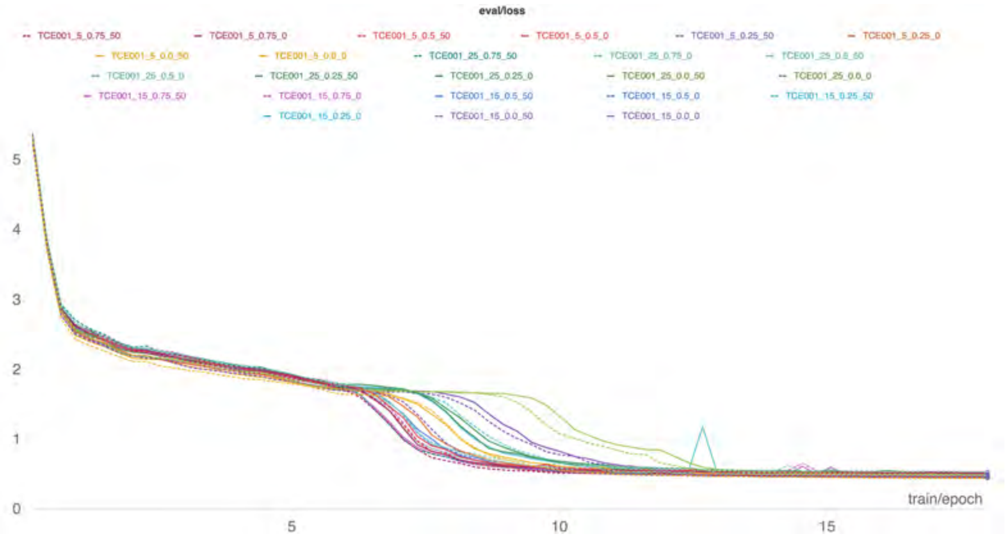

Figure C.1: Eval loss during pre-training, each line averages over 5 models with one and the same profile (number of authors, background ratio, reach threshold).

D Self-Training

Algorithm 2: Self-Training Loop

```
Input: max_steps = 600; num_texts = 200; decoding_parameters;
for step = 1 to max_steps do
    sen_list ← ∅ ;
    // 1.  generate texts and retain well-formed sentences
    for t = 1 to num_texts do
        // 1.1.  prompt construction
        prompt ← construct prompt with sentences the model believes to be true ;
        // 1.2.  text generation
        texts, scores ← query model with prompt (text completion task, beam sampling with
          decoding_parameters) ;
        // 1.3.  split texts and retain well-formed sentences
        sentences ← split texts into sub-sequences of length 3 ;
        sentences ← remove mis-formed sentences from sentences ;
        sen_list ← append sentences and corresp. scores to sen_list
    end
    // 2.  filter well-formed sentences
    sen_list ← remove sentences originating from texts with less than 6 well-formed sentences ;
    sen_list ← remove sentences originating from texts with score below 85th score percentile ;
    // 3.  construct denoising training data
    train_data ← mask predicate letter in each sentence from sen_list ;
    // 4.  train model
    model ← train model on train_data for 1 epoch
end
```

Algorithm 3: Prompt Construction

```
Input: len_prompt = 3 // number of sentences
prompt ← ∅ ;
a_1, a_2, a_3, a_4 ← sample (len_prompt + 1) tokens from domain D ;
for i = 1 to len_prompt do
    s ← a_i R a_{i+1} ;
    s̄ ← a_i S a_{i+1} ;
    if BEL_M(s) > BEL_M(s̄) then
        prompt ← append s to prompt ;
    else
        prompt ← append s̄ to prompt ;
    end
end
return prompt
```

To generate text, we use beam sampling decoding as implemented by Wolf et al. [2020] with the following parameters:

number of beams	5
number of return sequences	5
do_sample	True
top_p	.7
max_length	60
no_repeat_ngram_size	3

E Supplementary Results

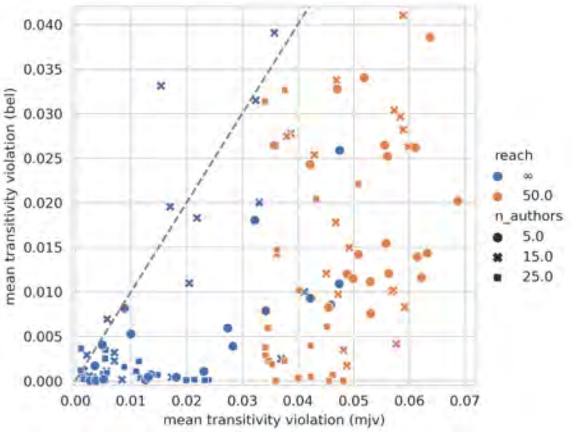

Figure E.1: Initial transitivity violation (mean) according to degrees of belief (y-axis) and majority-vote ratios (x-axis).

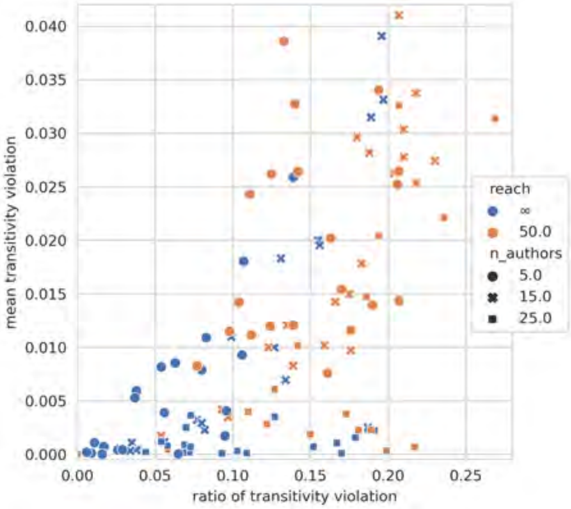

Figure E.2: Initial violations of transitivity constraint.

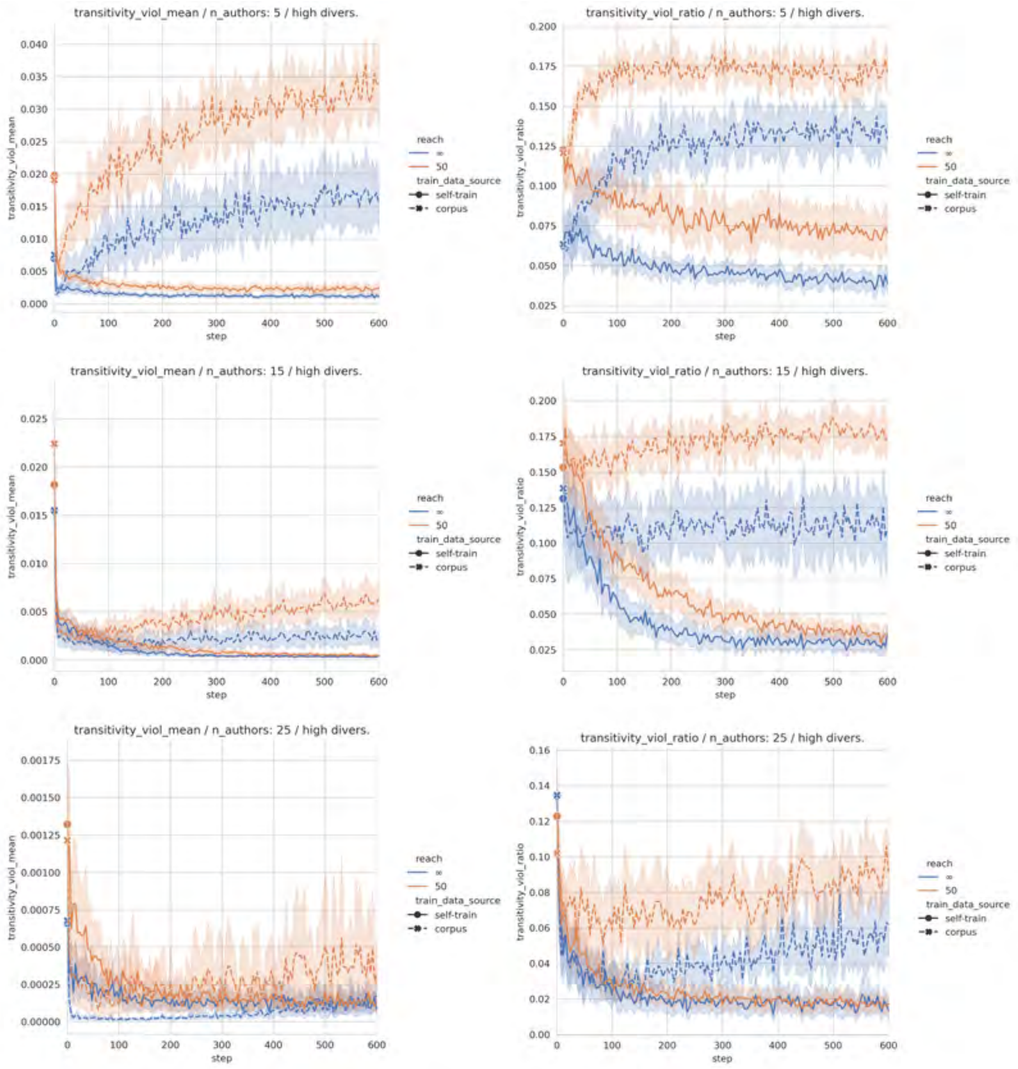

Figure E.3: Transitivity violation evolution during self-training, compared to baseline where model is trained with texts from corpus (`kendall_tau < 0.055`).

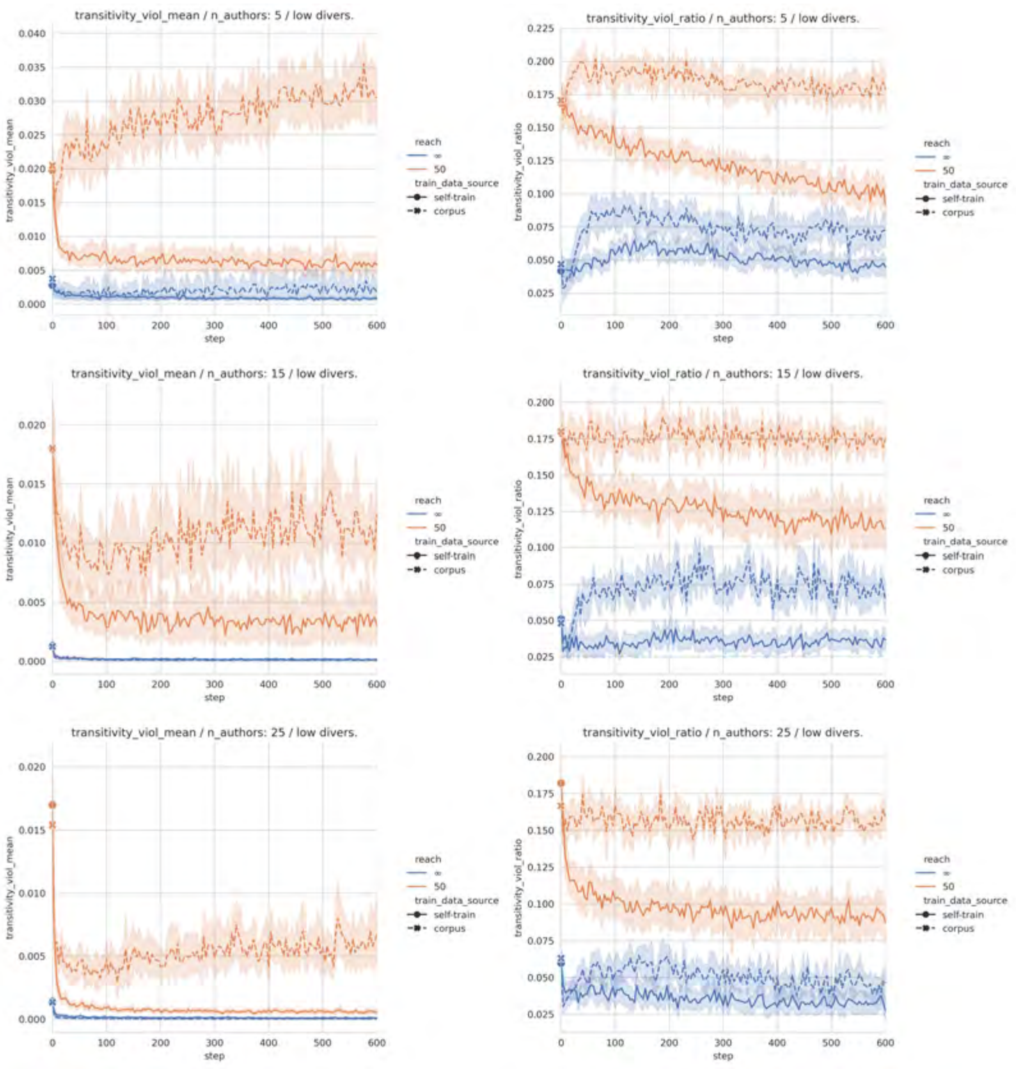

Figure E.4: Transitivity violation evolution during self-training, compared to baseline where model is trained with texts from corpus (`kendall_tau > 0.055`).

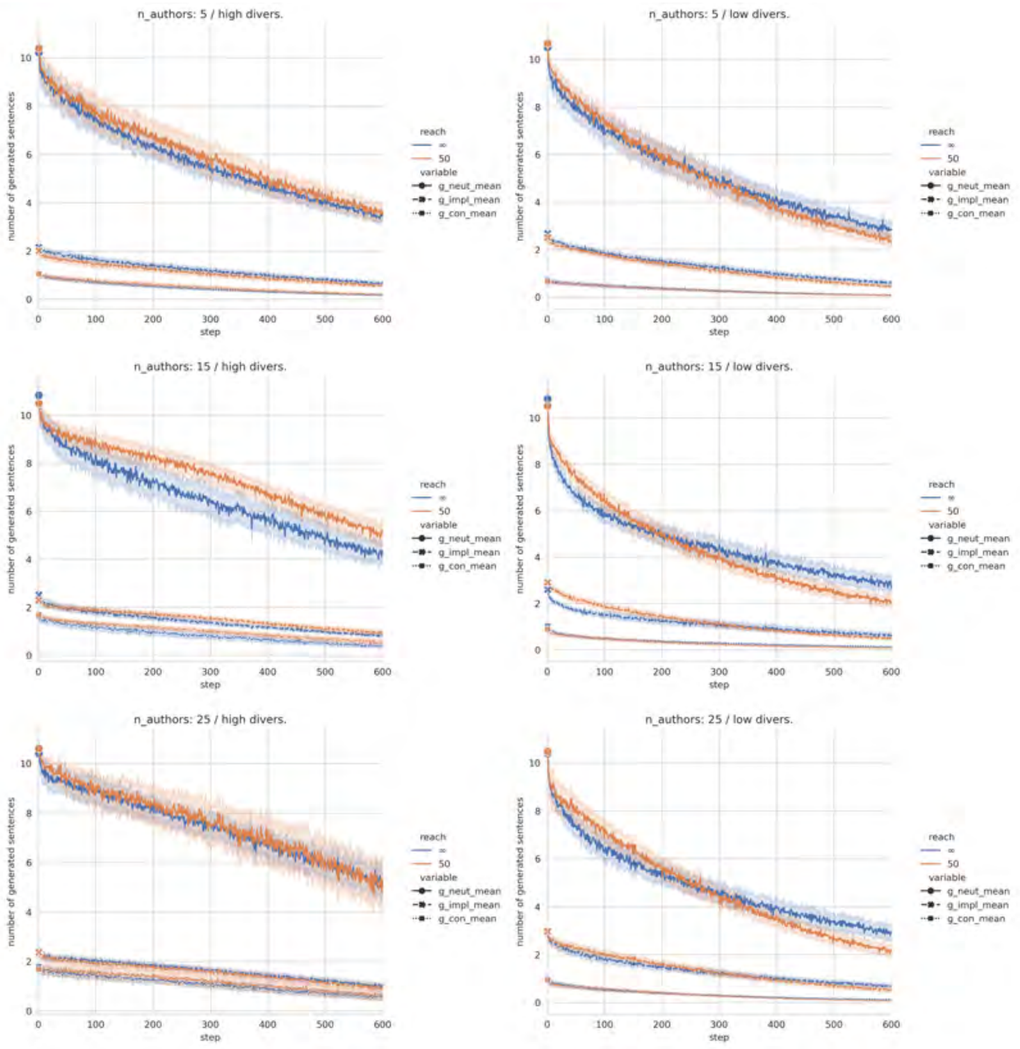

Figure E.5: Number of well-formed sentences per generated text that are neutral, are entailed, or contradict previous text sequence.

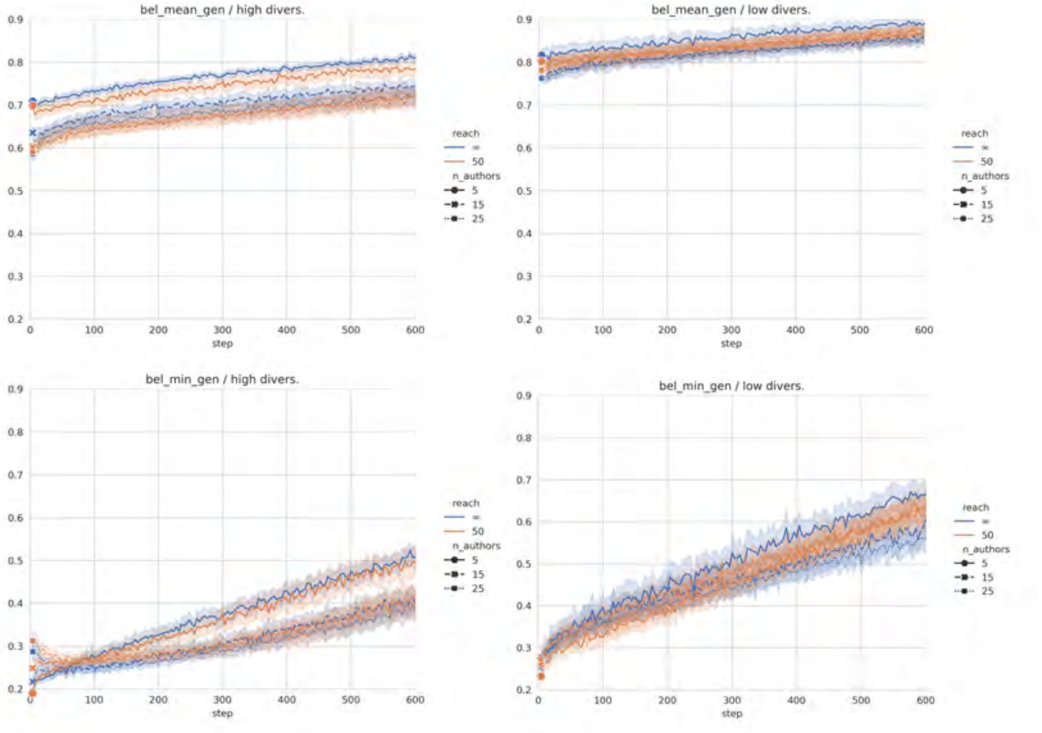

Figure E.6: Mean and minimum degree of belief in sentences in self-generated texts during self-training.

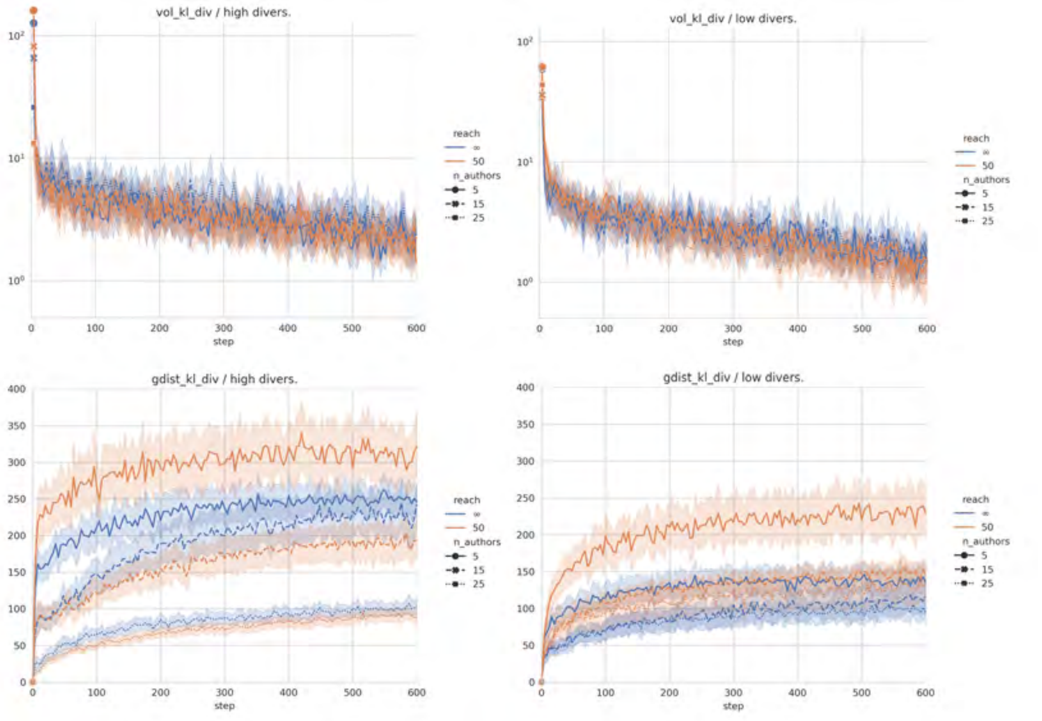

Figure E.7: Volatility and global distance to initial beliefs.
